# Equivalence of variance components between standard and recursive genetic models using LDL′ transformations

**DOI:** 10.1186/s12711-024-00901-x

**Published:** 2024-05-02

**Authors:** Luis Varona, David López-Carbonell, Houssemeddine Srihi, Carlos Hervás-Rivero, Óscar González-Recio, Juan Altarriba

**Affiliations:** 1 Instituto Agroalimentario de Aragón (IA2), Universidad de Zaragoza, c/Miguel Servet 177, 50013 Saragossa, Spain; 2 Instituto Nacional de Investigación y Tecnología Agraria y Alimentaria (INIA-CSIC), 28040 Madrid, Spain

## Abstract

**Background:**

Recursive models are a category of structural equation models that propose a causal relationship between traits. These models are more parameterized than multiple trait models, and they require imposing restrictions on the parameter space to ensure statistical identification. Nevertheless, in certain situations, the likelihood of recursive models and multiple trait models are equivalent. Consequently, the estimates of variance components derived from the multiple trait mixed model can be converted into estimates under several recursive models through LDL′ or block-LDL′ transformations.

**Results:**

The procedure was employed on a dataset comprising five traits (birth weight—BW, weight at 90 days—W90, weight at 210 days—W210, cold carcass weight—CCW and conformation—CON) from the Pirenaica beef cattle breed. These phenotypic records were unequally distributed among 149,029 individuals and had a high percentage of missing data. The pedigree used consisted of 343,753 individuals. A Bayesian approach involving a multiple-trait mixed model was applied using a Gibbs sampler. The variance components obtained at each iteration of the Gibbs sampler were subsequently used to estimate the variance components within three distinct recursive models.

**Conclusions:**

The LDL′ or block-LDL′ transformations applied to the variance component estimates achieved from a multiple trait mixed model enabled inference across multiple sets of recursive models, with the sole prerequisite of being likelihood equivalent. Furthermore, the aforementioned transformations simplify the handling of missing data when conducting inference within the realm of recursive models.

## Background

Recursive models are a type of structural equation model that propose a causal relationship between traits [1]. They were originally introduced to the field of animal breeding by Gianola and Sorensen [2]. Subsequently, they gained widespread usage in several applications [3–5].

It has been demonstrated that standard multiple trait models [6] and full recursive models yield equivalent likelihoods [7]. However, recursive models are more parameterized. Therefore, implementing recursive models requires imposing specific restrictions on the parameter space to ensure statistical identification [2]. Typically, two strategies have been used for this purpose. The first strategy involves imposing restrictions on the (co)variance component matrices [7], while the second strategy involves imposing constraints on the linear combinations of the explanatory variables [2]. In animal breeding, the most commonly used restriction in recursive models assumes that all nongenetic relationships arise solely from causality. This assumption leads to the imposition of a diagonal residual (co)variance matrix.

Although standard multiple trait models and fully recursive models exhibit equivalent likelihoods, the implementation of a recursive model typically requires either that all individuals have phenotypic information for all recorded traits or that a model-specific data-augmentation algorithm [8] is used to handle missing records. In contrast, multiple trait models do not require a complete dataset, and numerous software options for likelihood or Bayesian inference are available [9–11].

In this study, we adopted the approach suggested by Varona and González-Recio [12], which involves employing LDL′ and block-LDL′ transformations of the residual (co)variance matrices. These transformations, based on specific assumptions, enable conversion of the results of (co)variance component estimation within a multiple trait model into several recursive mixed models, providing alternative interpretations of the data. The objective was to demonstrate the implementation of this transformation within a Gibbs sampler algorithm on growth traits from the Spanish Pirenaica beef cattle population.

## Methods

### Dataset

The phenotypic dataset used in this study consisted of five traits (birth weight—BW, 90-day weight—W90, 210-day weight—W210, cold carcass weight—CCW and conformation—CONF), derived from the standard genetic evaluation of the Pirenaica beef cattle breed. The database included 149,029 birth weights (BW), 59,578 weights within the interval between 60 and 120 days of age (W90), 46,550 weights within 170 and 250 days of age (W210), 52,110 cold carcass weights (CCW), and 50,459 conformation ratings (CONF) on the SEUROP scale [13]. The SEUROP scale was transformed into a numerical scale ranging from 1 (P−) to 18 (S+). For a detailed description of the phenotypic data, please refer to Table 1.

**Table 1:** Number of data, mean and standard deviation for the analysed traits

Trait	N	Mean	SD
BW	149,029	41.12	4.49
W90	59,578	134.46	41.70
W210	46,550	250.71	59.21
CCW	52,110	300.29	55.91
CONF	50,459	11.86	1.38

*N* number of records, *SD* standard deviation, *BW* birth weight, *W90* weight at 90 days, *W210* weight at 210 days, *CCW* cold carcass weight, *CONF* conformation

**Fig. 1:**
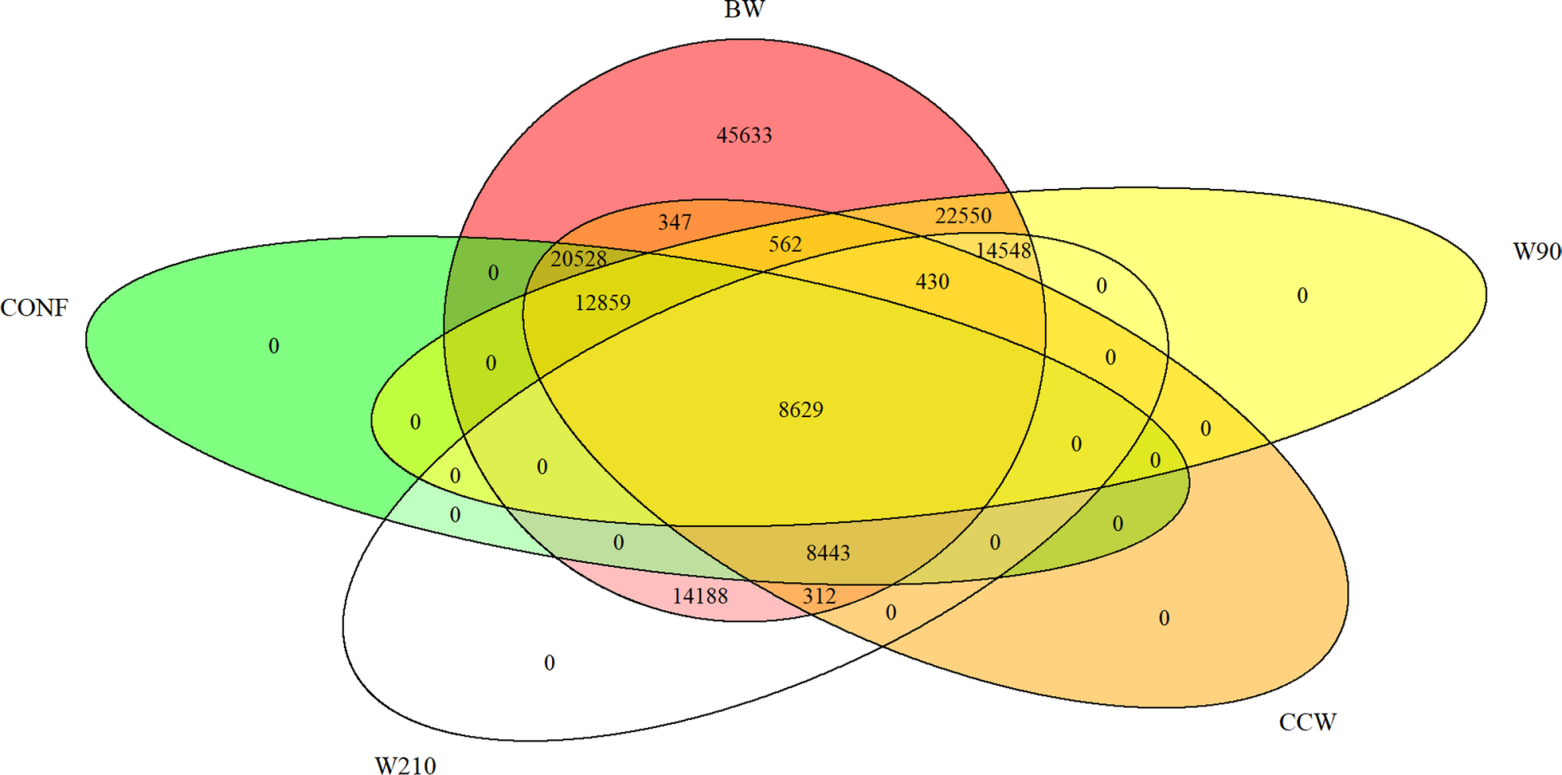
Distribution of recorded phenotyped across individuals. *BW* birth weight, *W90* weight at 90 days, *W210* weight at 210 days, *CCW* cold carcass weight, *CONF* conformation

Only 8629 individuals had recorded data for all five traits. Among the recorded combinations of traits, the most common were BW–W90 (22,550 individuals), BW–CCW–CONF (20,528 individuals), BW–W90–W210 (14,548 individuals), BW–W210 (14,188 individuals), BW–W90–CCW–CONF (12,859 individuals), and BW–W210–CCW–CONF (8443 individuals). A detailed description of the distribution of recorded phenotypes among the individuals is displayed in Fig. 1.

### Model for analysis

#### Multiple trait model

The data were analysed using the standard multiple trait model (SM) within a Bayesian approach by using a Gibbs sampler [14]. The statistical model was:

1 $${\mathbf{y}}_{{\text{i}}}={\mathbf{X}}_{{\text{i}}}\mathbf{b}+{\mathbf{u}}_{{\text{i}}}+{\mathbf{e}}_{{\text{i}}}.$$

In this model, the vector of fixed effects, denoted as $\mathbf{b}$, incorporates various systematic effects, such as a covariate with the age of recording for W90, W210, CCW, and CONF, sex, age of dam (6 levels), and herd-year-season (4824 levels). The variables ${\mathbf{y}}_{{\text{i}}}$, ${\mathbf{u}}_{{\text{i}}}$ and ${\mathbf{e}}_{\mathbf{i}}$ are 5 × 1 vectors of the phenotypic records, additive genetic effects, and residuals, respectively, associated with the ${\text{i}}$th recorded individual for the five traits. It is important to note that the ${\mathbf{y}}_{\mathbf{i}}$ vector may be composed of observed (${\mathbf{y}}_{{\text{i}}}^{{\text{O}}}$) and missing records (${\mathbf{y}}_{{\text{i}}}^{{\text{M}}}$). Missing records had to be augmented under a data-augmentation step [8] within the Gibbs sampler. ${\mathbf{X}}_{\mathbf{i}}$ is the corresponding incidence matrix. The vectors of breeding values $\left({\mathbf{u}}^{\mathbf{\prime}}=\left\{{\mathbf{u}}_{\mathbf{1}}^{\mathbf{\prime}},{\mathbf{u}}_{\mathbf{2}}^{\mathbf{\prime}},\ldots , {\mathbf{u}}_{\mathbf{n}}^{\mathbf{\prime}},{\mathbf{u}}_{\mathbf{n}+\mathbf{1}}^{\mathbf{\prime}},\ldots , {\mathbf{u}}_{\mathbf{s}}^{\mathbf{\prime}}\right\}\right)$ and residual effects $\left({\mathbf{e}}^{\mathbf{\prime}}=\left\{{\mathbf{e}}_{\mathbf{1}}^{\mathbf{\prime}},{\mathbf{e}}_{\mathbf{2}}^{\mathbf{\prime}},\dots , {\mathbf{e}}_{\mathbf{n}}^{\mathbf{\prime}}\right\}\right)$ for the observed and missing records are assumed to follow a multivariate Gaussian distribution:

2 $${\mathbf{u}}\sim {\text{N}}\left( {{\mathbf{0}},{\mathbf{A}} \otimes {\mathbf{G}}} \right)\;{\text{and}}\;{\mathbf{e}}\sim {\text{N}}\left( {{\mathbf{0}},{\mathbf{I}} \otimes {\mathbf{R}}} \right).$$

Here, ${\text{n}}$ denotes the number of multivariate phenotypic records, ${\text{s}}$ represents the number of individuals, including both those with (${\text{n}}$) and without (${\text{s}}-{\text{n}}$) recorded phenotypes, and $\mathbf{G}$ and $\mathbf{R}$ are ${\text{m}}\times {\text{m}}$ (${\text{m}}=5$) matrices, with $\mathbf{G}$ representing the genetic (co)variances and $\mathbf{R}$ representing the residual (co)variances. $\mathbf{I}$ is an identity matrix of the corresponding order, and $\mathbf{A}$ represents the numerator relationship matrix, which was computed from a pedigree of 343,753 individual-sire-dam entries. This SM was implemented under a Bayesian approach through the gibbsf90+ software [9]. The Gibbs sampler involves sampling from the full conditional distributions of all the unknowns in the model. Specifically, the full conditional distributions of the elements of ${\mathbf{y}}_{{\text{i}}}^{{\text{M}}}$, $\mathbf{b}$ and ${\mathbf{u}}_{{\text{i}}}$ were Gaussian, whereas the full conditional distributions for $\mathbf{R}$ and $\mathbf{G}$ followed an inverse Wishart distribution [15]. The Gibbs sampler [14] was used with 500,000 iterations after discarding the initial 100,000 iterations to ensure convergence to the posterior distribution. Convergence was checked with the coda software [16] and the effective size for the additive genetic variances for BW, W90, W210, CCW and CONF were 2771.46, 1282.81, 853.35, 663.84 and 859.53, respectively.

#### Recursive models

The SM can be transformed into a recursive mixed model (RM) by multiplying all the terms by the matrix ${\varvec{\Lambda}}$. This results in the following model:

3 $${\varvec{\Lambda}}{\mathbf{y}}_{{\text{i}}}={{\varvec{\Lambda}}\mathbf{X}}_{{\text{i}}}\mathbf{b}+{{\varvec{\Lambda}}\mathbf{u}}_{{\text{i}}}+{{\varvec{\Lambda}}\mathbf{e}}_{{\text{i}}}={{\varvec{\Lambda}}\mathbf{X}}_{{\text{i}}}\mathbf{b}+{\mathbf{u}}_{\mathbf{i}}^{\mathbf{*}}+{\mathbf{e}}_{\mathbf{i}}^{\mathbf{*}}.$$

Here, ${\varvec{\Lambda}}$ is a 5 × 5 matrix representing the recursive parameters. It contains 1s on the diagonal and, in some or all the elements below the diagonal, minus the recursive effects of the ${\text{i}}$th trait on the ${\text{j}}$th trait (− ${\uplambda }_{{\text{i}}\to {\text{j}}}$). The terms ${\mathbf{u}}_{\mathbf{i}}^{\mathbf{*}}={{\varvec{\Lambda}}\mathbf{u}}_{{\text{i}}}$ and ${\mathbf{e}}_{\mathbf{i}}^{\mathbf{*}}={{\varvec{\Lambda}}\mathbf{e}}_{{\text{i}}}$ are the 5 × 1 vectors of additive genetic effects and residuals for the ${\text{i}}$th multivariate record under the recursive model, with:

4 $${\mathbf{u}}^{*} \sim {\text{N}}\left( {{\mathbf{0}},{\mathbf{A}} \otimes {\mathbf{G}}^{*} } \right)\;{\text{and}}\;{ }{\mathbf{e}}^{*} \sim {\text{N}}\left( {{\mathbf{0}},{\mathbf{I}} \otimes {\mathbf{R}}^{*} } \right).$$

Furthermore, ${\mathbf{G}}^{\mathbf{*}}={\varvec{\Lambda}}\mathbf{G}{{\varvec{\Lambda}}}^{\mathbf{\prime}}$ and ${\mathbf{R}}^{\mathbf{*}}={\varvec{\Lambda}}\mathbf{R}{{\varvec{\Lambda}}}^{\mathbf{\prime}}$*.* It is worth noting that recursive models require certain restrictions, such as imposing constraints on the (co)variance matrices [7] or incorporating instrumental auxiliary variables that exclusively affect the dependent traits through the independent traits [2]. The most common of these restrictions involves setting the residual covariance between traits linked by a recursive parameter to zero. In other words, this restriction assumes that traits can be interconnected either through a causal dependency or via residual covariance between them.

In this study, as a post-computational step, we applied LDL′ and block-LDL′ transformations to the $\mathbf{R}$ (co)variance matrices obtained at each iteration of the Gibbs sampler iteration. These matrices were computed using residuals for both observed and augmented phenotypes. This led to the emergence of three distinct scenarios, each portraying varying causal relationships between the traits.

**First scenario**: In the first scenario, traits were organized in the recursive model depending on their recording time (BW → W90 → W210 → CCW → CONF), with the assumption that each preceding trait exerted a causal influence on all subsequent traits (i.e. ${\varvec{\Lambda}}$ does not contain any zero elements below the diagonal). In the recursive model, the ${\mathbf{R}}^{\boldsymbol{*}}$ matrix is commonly assumed to be diagonal. Consequently, $\mathbf{R}$ can be factorized using LDL′ decomposition ($\mathbf{R}=\mathbf{L}\mathbf{D}\mathbf{L}^{\prime}$), where $\mathbf{D}$ corresponds to the ${\mathbf{R}}^{\boldsymbol{*}}$ matrix and $\mathbf{L}$ represents ${{\varvec{\Lambda}}}^{-1}$. Moreover, the ${\mathbf{G}}^{\mathbf{*}}$ matrix is obtained as ${\mathbf{G}}^{\mathbf{*}}={\varvec{\Lambda}}\mathbf{G}{{\varvec{\Lambda}}}^{\prime}={\mathbf{L}}^{-\mathbf{1}}\mathbf{G}{\mathbf{L}}^{-\mathbf{1}\mathbf{\prime}}$.

**Second scenario**: In this scenario, the traits were categorized into two groups. The first group consisted of traits recorded on the farm (BW, W90, and W210), while the second group encompassed traits recorded at the slaughterhouse (CCW and CONF). We used a block LDL′ transformation of the $\mathbf{R}$ matrix, as follows:

5 $$\begin{aligned} {\mathbf{R}} & = \left[ {\begin{array}{*{20}c} {\mathbf{E}} & {{\mathbf{B^{\prime}}}} \\ {\mathbf{B}} & {\mathbf{C}} \\ \end{array} } \right] \\ & = \left[ {\begin{array}{*{20}c} {\mathbf{I}} & {\mathbf{0}} \\ {{\mathbf{BE}}^{ - 1} } & {\mathbf{I}} \\ \end{array} } \right]\left[ {\begin{array}{*{20}c} {\mathbf{E}} & {\mathbf{0}} \\ {\mathbf{0}} & {{\mathbf{C}} - {\mathbf{BE}}^{ - 1} {\mathbf{B^{\prime}}}} \\ \end{array} } \right]\left[ {\begin{array}{*{20}c} {\mathbf{I}} & {{\mathbf{E}}^{{ - 1}} {\mathbf{B^{\prime}}}} \\ {\mathbf{0}} & {\mathbf{I}} \\ \end{array} } \right] = {{\varvec{\Lambda}}}^{ - 1} {\mathbf{R}}^{*} {{\varvec{\Lambda}}}^{{ - 1{\prime}}} , \\ \end{aligned}$$

where

6 $$\mathbf{E}=\left[\begin{array}{ccc}{\upsigma }_{{\text{e}}({\text{BW}})}^{2}& {\upsigma }_{{\text{e}}({\text{BW}}-{\text{W}}90)}& {\upsigma }_{{\text{e}}({\text{BW}}-{\text{W}}210)}\\ {\upsigma }_{{\text{e}}({\text{BW}}-{\text{W}}90)}& {\upsigma }_{{\text{e}}({\text{W}}90)}^{2}& {\upsigma }_{{\text{e}}({\text{W}}90-{\text{W}}210)}\\ {\upsigma }_{{\text{e}}({\text{BW}}-{\text{W}}210)}& {\upsigma }_{{\text{e}}({\text{W}}90-{\text{W}}210)}& {\upsigma }_{{\text{e}}({\text{W}}210)}^{2}\end{array}\right],$$

7 $$\mathbf{B}=\left[\begin{array}{ccc}{\upsigma }_{{\text{e}}({\text{BW}}-{\text{CCW}})}& {\upsigma }_{{\text{e}}({\text{W}}90-{\text{CCW}})}& {\upsigma }_{{\text{e}}({\text{W}}210-{\text{CCW}})}\\ {\upsigma }_{{\text{e}}({\text{BW}}-{\text{CONF}})}& {\upsigma }_{{\text{e}}({\text{W}}90-{\text{CONF}})}& {\upsigma }_{{\text{e}}({\text{W}}210-{\text{CONF}})}\end{array}\right],$$

8 $$\mathbf{C}=\left[\begin{array}{cc}{\sigma }_{e(CCW)}^{2}& {\sigma }_{e(CCW-CONF)}\\ {\sigma }_{e(CCW-CONF)}& {\sigma }_{e(CONF)}^{2}\end{array}\right],$$

and, as before, ${\mathbf{G}}^{\mathbf{*}}={\varvec{\Lambda}}\mathbf{G}{{\varvec{\Lambda}}}^{\prime}$. Note that ${\sigma }_{e(X-Y)}$ represents the residual covariance between traits X and Y, and ${\sigma }_{e(X)}^{2}$ is the residual variance of trait X, with X = Y = {BW, W90, W210, CCW, CONF}.

**Third scenario**: In this scenario, we defined three time points: BW → (W90–W210) → (CCW–CONF). To achieve this, we can use sequential block-LDL′ decompositions:

First LDL′ decomposition:

9 $$\begin{aligned} {\mathbf{R}} & = {\mathbf{R}}_{\mathbf{1}} = \left[ {\begin{array}{*{20}c} {{\mathbf{E}}_{\mathbf{1}} } & {{\mathbf{B}}_{\mathbf{1}}^{\prime} } \\ {{\mathbf{B}}_{\mathbf{1}} } & {{\mathbf{C}}_{\mathbf{1}} } \\ \end{array} } \right] \\ & = \left[ {\begin{array}{*{20}c} {\mathbf{I}} & {0} \\ {{\mathbf{B}}_{\mathbf{1}} {\mathbf{E}}_{\mathbf{1}}^{-\mathbf{1}} } & {\mathbf{I}} \\ \end{array} } \right]\left[ {\begin{array}{*{20}c} {{\mathbf{E}}_{\mathbf{1}} } & {\mathbf{0}} \\ {\mathbf{0}} & {{\mathbf{C}}_{\mathbf{1}} - {\mathbf{B}}_{\mathbf{1}} {\mathbf{E}}_{\mathbf{1}}^{ - \mathbf{1}} {\mathbf{B}}_{\mathbf{1}}^{\prime} } \\ \end{array} } \right]\left[ {\begin{array}{*{20}c} {\mathbf{I}} & {{\mathbf{E}}_{1}^{ - 1} {\mathbf{B}}_{1}^{\prime} } \\ {\mathbf{0}} & {\mathbf{I}} \\ \end{array} } \right] \\ & = {{\varvec{\Lambda}}}_{\mathbf{1}}^{ - 1} {\mathbf{R}}_{\mathbf{2}} {{\varvec{\Lambda}}}_{\mathbf{1}}^{{ - 1^{\prime}}} . \\ \end{aligned}$$

In this step, the traits were divided in two groups. The first group included only BW, while the second comprised W90, W210, CCW and CONF. Therefore, ${\mathbf{E}}_{\mathbf{1}}$ was a 1 × 1 matrix, ${\mathbf{B}}_{\mathbf{1}}$ was a 4 × 1 matrix and ${\mathbf{C}}_{\mathbf{1}}$ was a 4 × 4 matrix. The output of this step was an auxiliary matrix (${\mathbf{R}}_{\mathbf{2}}$) that was further LDL′ decomposed in the next step.

Second LDL′ decomposition:

10 $$\begin{aligned} {\mathbf{R}}_{\mathbf{2}} & = \left[ {\begin{array}{*{20}c} {{\mathbf{E}}_{\mathbf{2}} } & {{\mathbf{B}}_{\mathbf{2}}^{\prime} } \\ {{\mathbf{B}}_{\mathbf{2}} } & {{\mathbf{C}}_{\mathbf{2}} } \\ \end{array} } \right] \\ & = \left[ {\begin{array}{*{20}c} {\mathbf{I}} & \mathbf{0} \\ {{\mathbf{B}}_{\mathbf{2}} {\mathbf{E}}_{\mathbf{2}}^{ - \mathbf{1}} } & {\mathbf{I}} \\ \end{array} } \right]\left[ {\begin{array}{*{20}c} {{\mathbf{E}}_{\mathbf{2}} } & \mathbf{0} \\ \mathbf{0} & {{\mathbf{C}}_{\mathbf{2}} - {\mathbf{B}}_{\mathbf{2}} {\mathbf{E}}_{\mathbf{2}}^{ - \mathbf{1}} {\mathbf{B}}_{\mathbf{2}}^{\prime} } \\ \end{array} } \right]\left[ {\begin{array}{*{20}c} {\mathbf{I}} & {{\mathbf{E}}_{\mathbf{2}}^{{ - \mathbf{1}}} {\mathbf{B}}_{\mathbf{2}}^{\prime} } \\ \mathbf{0} & {\mathbf{I}} \\ \end{array} } \right] \\ & = {{\varvec{\Lambda}}}_{\mathbf{2}}^{ - 1} {\mathbf{R}}^{*} {{\varvec{\Lambda}}}_{\mathbf{2}}^{{ -1^{\prime}}} . \\ \end{aligned}$$

Here, the first group was composed of BW, W90 and W210 and the second group of CCW and CONF, respectively. Therefore, ${\mathbf{E}}_{\mathbf{2}}$ was a 3 × 3 matrix, ${\mathbf{B}}_{\mathbf{2}}$ was a 2 × 3 matrix and ${\mathbf{C}}_{\mathbf{2}}$ was a 2 × 2 matrix.

Finally, $$\mathbf{R}={{{\varvec{\Lambda}}}_{\mathbf{1}}^{-1}{\varvec{\Lambda}}}_{\mathbf{2}}^{-1}{\mathbf{R}}^{\mathbf{*}}{{{\varvec{\Lambda}}}_{\mathbf{2}}^{-1}}^{\prime}{{{\varvec{\Lambda}}}_{\mathbf{1}}^{-1}}^{\prime}={{\varvec{\Lambda}}}^{-1}{\mathbf{R}}^{\mathbf{*}}{{\varvec{\Lambda}}}^{-1\mathbf{\prime}}$$ and $${\mathbf{G}}^{\mathbf{*}}={\varvec{\Lambda}}\mathbf{G}{{\varvec{\Lambda}}}^{\prime}$$.

## Results

### Multiple trait model (SM)

The posterior mean estimates (and their standard deviations) of additive genetic variances, covariances, and correlations derived from the multiple trait model are in Table 2, while the corresponding values for residual variances, covariances, and correlations are in Table 3.

**Table 2:** Posterior mean (and posterior deviation) of the additive genetic variances (diagonal), additive genetic covariances (upper diagonal) and additive genetic correlations (lower diagonal) from the standard multivariate model

	BW	W90	W210	CCW	CONF
BW	4.85 (0.10)	9.85 (0.67)	16.50 (1.10)	20.70 (1.24)	− 0.01 (0.05)
W90	0.26 (0.02)	297.46 (11.33)	288.29 (10.68)	171.06 (10.69)	1.69 (0.37)
W210	0.30 (0.02)	0.67 (0.02)	629.70 (23.47)	347.78 (17.54)	− 0.76 (0.63)
CCW	0.40 (0.02)	0.43 (0.02)	0.60 (0.02)	540.36 (22.87)	6.10 (0.56)
CONF	− 0.00 (0.02)	0.11 (0.02)	− 0.03 (0.03)	0.28 (0.02)	0.86 (0.03)

*BW* birth weight, *W90* weight at 90 days, *W210* weight at 210 days, *CCW* cold carcass weight, *CONF* conformation

**Table 3:** Posterior mean (and posterior deviation) of the residual variances (diagonal), residual covariances (upper diagonal) and residual correlations (lower diagonal) from the standard multivariate model

	BW	W90	W210	CCW	CONF
BW	8.93 (0.07)	11.85 (0.49)	14.91 (0.84)	13.64 (0.91)	0.37 (0.03)
W90	0.19 (0.01)	452.24 (5.86)	452.16 (8.54)	228.91 (8.03)	1.16 (0.28)
W210	0.14 (0.01)	0.60 (0.01)	1268.18 (17.31)	482.62 (13.31)	5.06 (0.47)
CCW	0.16 (0.01)	0.38 (0.01)	0.48 (0.01)	791.88 (15.62)	10.33 (0.38)
CONF	0.15 (0.01)	0.07 (0.02)	0.18 (0.01)	0.46 (0.01)	0.65 (0.02)

*BW* birth weight, *W90* weight at 90 days, *W210* weight at 210 days, *CCW* cold carcass weight, *CONF* conformation

The posterior mean estimates (and their standard deviations) of the heritabilities were: 0.352 (0.006) for BW, 0.397 (0.009) for W90, 0.332 (0.011) for W210, 0.406 (0.015) for CCW, and 0.569 (0.015) for CONF. These results align closely with those obtained in a previous study conducted on the same population [13] and fall within the range of values observed in other beef cattle populations [17].

#### First scenario

The first scenario assumes a fully recursive model in which traits are recorded sequentially (BW → W90 → W210 → CCW → CONF), positing that all traits influence those that are recorded later. Following the implementation of the LDL′ decomposition on the residual covariance matrix ($\mathbf{R}$) obtained from each iteration of the Gibbs sampler, the posterior mean (and standard deviation) for the residual variances under the recursive model are 8.93 (0.07) for BW, 436.48 (5.67) for W90, 814.82 (13.08) for W210, 591.56 (13, 12) for CCW and 0.50 (0.02) for CONF. As expected, the residual variance of the dependent traits (W90, W210, CCW and CONF) was smaller than that in the SM analysis, as a portion of the residual variability was explained by the recursive parameters. Furthermore, the posterior distribution statistics of the additive genetic variances, covariances and correlations from the recursive model assumed under the first scenario are in Table 4.

**Table 4:** Posterior mean (and posterior deviation) of the additive genetic variances (diagonal), additive genetic covariances (upper diagonal) and additive genetic correlations (lower diagonal) within the recursive model in the first scenario

	BW	W90	W210	CCW	CONF
BW	4.85 (0.10)	3.42 (0.86)	5.02 (1.26)	10.10 (1.58)	− 0.37 (0.06)
W90	0.09 (0.02)	279.91 (8.23)	− 16.55 (12.81)	8.20 (13.42)	1.09 (0.49)
W210	0.12 (0.03)	− 0.05 (0.04)	346.68 (16.94)	64.63 (15.86)	− 5.12 (0.66)
CCW	0.25 (0.04)	0.03 (0.03)	0.19 (0.04)	348.21 (18.69)	0.63 (0.46)
CONF	− 0.19 (0.03)	0.07 (0.03)	− 0.30 (0.04)	0.03 (0.04)	0.81 (0.03)

*BW* birth weight, *W90* weight at 90 days, *W210* weight at 210 days, *CCW* cold carcass weight, *CONF* conformation

**Fig. 2:**
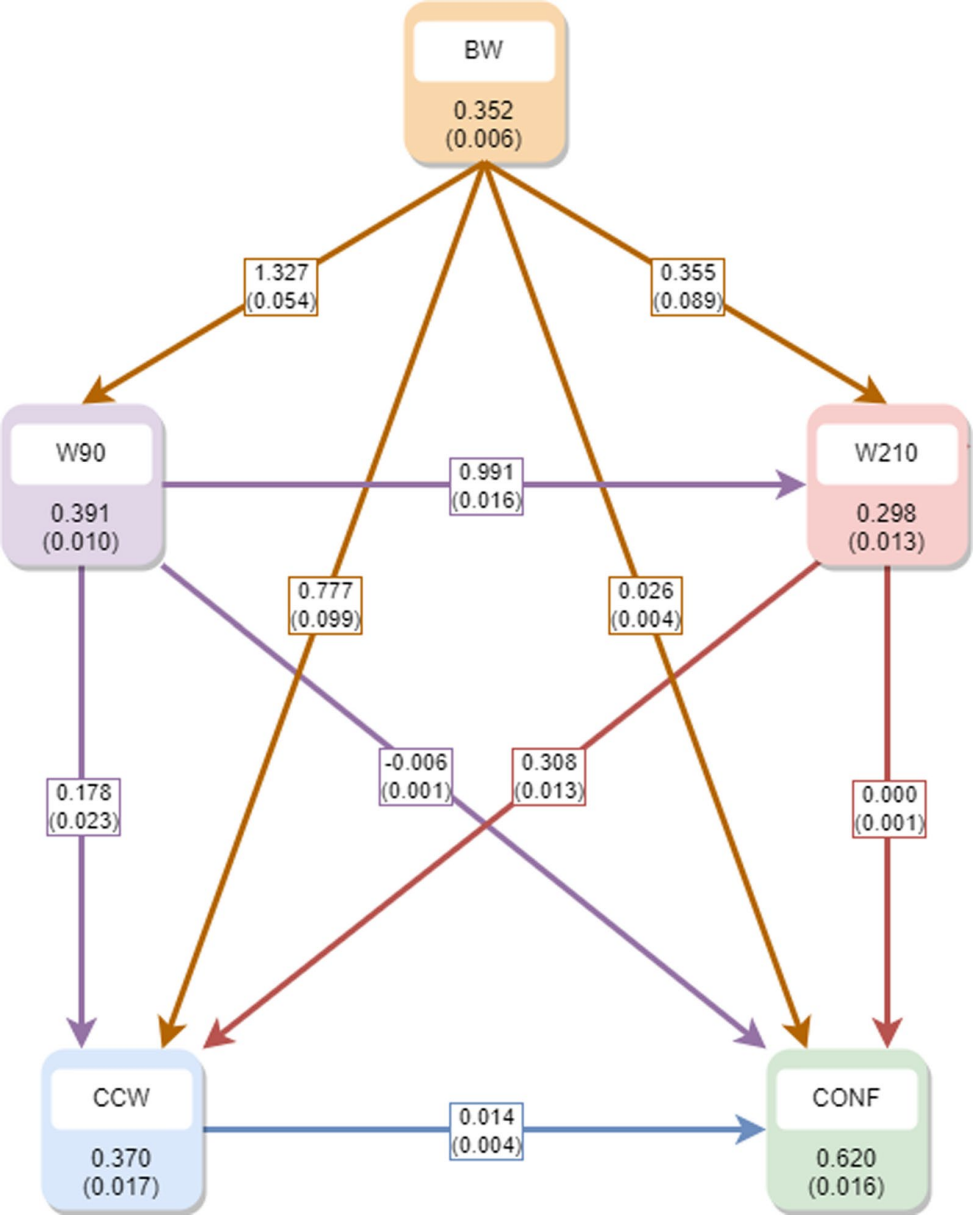
Posterior mean (and standard deviation) of the heritabilities (within the squares) and the recursive parameters (at the arrows) within the recursive model in the first scenario. *BW* birth weight, *W90* weight at 90 days, *W210* weight at 210 days, *CCW* cold carcass weight, *CONF* conformation

The additive genetic variances were smaller compared to those in the SM scenario, as they now solely represent the additive genetic variance resulting from genes that directly influence the dependent traits [18]. In addition, the posterior estimates of the genetic correlations approached zero more closely than in the SM scenario. This observation suggests that the majority of the additive genetic correlation between traits arises from the recursive relationships among them. Finally, Fig. 2 displays the posterior mean estimates (along with standard deviations) for heritabilities and recursive parameters in ${\varvec{\Lambda}}$ within the recursive model in the first scenarios.

It is noteworthy that all the recursive parameters exhibited positive values, which means that an enhancement in the traits recorded earlier corresponded to a phenotypic improvement in the traits recorded later in life. Furthermore, while the range of heritabilities appeared similar to that in the SM scenario, their interpretation differed. In this context, heritabilities represent the proportion of additive genetic variance associated with each trait while taking into account the influence of previously recorded traits [18, 19].

#### Second scenario

In this second scenario, the traits were divided into two distinct groups: one group consisted of traits that were recorded on the farm (BW, W90, and W210), while the other group included the traits recorded at the slaughterhouse (CCW and CONF). It posited a phenotypic influence of the traits in the first group on the traits in the second group. Table 5 presents the posterior mean estimates (along with the posterior standard deviations) for the additive genetic variances, covariances, and correlations within the recursive model in the second scenario.

**Table 5:** Posterior mean (and posterior deviation) of the additive genetic variances (diagonal), additive genetic covariances (upper diagonal) and additive genetic correlations (lower diagonal) within the recursive model in the second scenario

	BW	W90	W210	CCW	CONF
BW	4.85 (0.10)	9.85 (0.67)	16.50 (1.10)	10.09 (1.58)	− 0.23 (0.06)
W90	0.26 (0.02)	297.46 (11.33)	288.29 (10.68)	21.69 (13.79)	0.89 (0.54)
W210	0.30 (0.02)	0.67 (0.02)	629.70 (23.47)	89.72 (20.22)	− 3.42 (0.82)
CCW	0.25 (0.04)	0.07 (0.04)	0.19 (0.04)	348.21 (18.69)	5.32 (0.53)
CONF	− 0.11 (0.03)	0.05 (0.03)	− 0.14 (0.03)	0.30 (0.03)	0.89 (0.03)

*BW* birth weight, *W90* weight at 90 days, *W210* weight at 210 days, *CCW* cold carcass weight, *CONF* conformation

In addition, the results for the residual variances, covariances and correlations within the recursive model in the second scenario are in Table 6.

**Table 6:** Posterior mean (and posterior deviation) of the residual variances (diagonal), covariances (upper diagonal) and correlations (lower diagonal) within the recursive model in the second scenario

	BW	W90	W210	CCW	CONF
BW	8.93 (0.07)	11.85 (0.49)	14.91 (0.84)	–	–
W90	0.19 (0.01)	452.24 (5.86)	452.16 (8.54)	–	–
W210	0.14 (0.01)	0.60 (0.01)	1268.18 (17.31)	–	–
CCW	–	–	–	591.56 (13.12)	8.27 (0.36)
CONF	–	–	–	0.43 (0.01)	0.61 (0.02)

*BW* birth weight, *W90* weight at 90 days, *W210* weight at 210 days, *CCW* cold carcass weight, *CONF* conformation

**Fig. 3:**
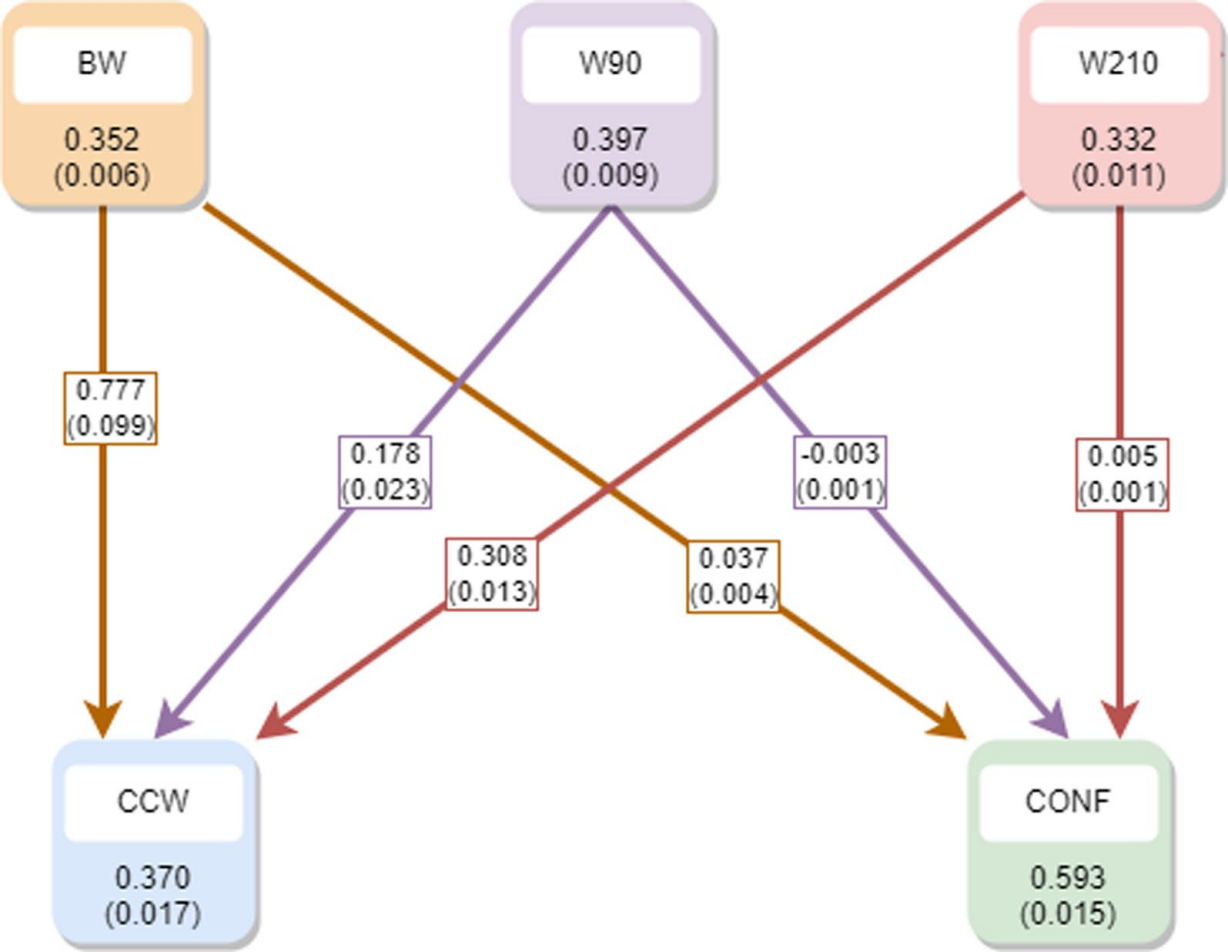
Posterior mean (and standard deviation) of the heritabilities (within the squares) and the recursive parameters (at the arrows) within the recursive model in the second scenario. *BW* birth weight, *W90* weight at 90 days, *W210* weight at 210 days, *CCW* cold carcass weight, *CONF* conformation

In this context, the additive and residual variances for the traits recorded on the farm (BW, W90, W210) remained identical to those in the SM model, with reductions observed only for the two dependent traits (CCW and CONF). In this scenario, the residual covariances (and correlations) between the first three traits (BW, W90, and W210) and CCW or CONF were set to zero. Furthermore, it is important to note that the variance components of CCW and CONF should be interpreted in the context of the traits recorded on the farm. The posterior mean estimates (with their standard deviations) for heritabilities and recursive parameters within the recursive model in the second scenario are illustrated in Fig. 3.

The estimates of the heritabilities for BW, W90, and W210 remain equal to those of the SM model, and the model only introduces recursive parameters between traits within the first group (BW, W90, and W210) on the traits recorded at the slaughterhouse (CONF and CCW).

#### Third scenario

In this last scenario, the traits were divided into three distinct groups: the first group consisted solely of BW, the second group comprised W90 and W210, and the third group was composed of CCW and CONF. The model posits that BW exerts a phenotypic influence on the traits in the second and third groups, while the traits in the second group influence those in the third group. In this scenario, the procedure involves resolving a sequential block LDL′ for the output of the variance components in each iteration of the Gibbs sampler with the SM model. The posterior mean estimates (and respective posterior standard deviations) for the additive genetic variances, covariances, and correlations within the recursive model in the third scenario are in Table 7.

**Table 7:** Posterior mean (and posterior deviation) of the additive genetic variances (diagonal), additive genetic covariances (upper diagonal) and additive genetic correlations (lower diagonal) within the recursive model in the third scenario

	BW	W90	W210	CCW	CONF
BW	4.85 (0.10)	3.42 (0.86)	8.40 (1.42)	10.10 (1.58)	− 0.23 (0.06)
W90	0.09 (0.02)	279.91 (8.23)	260.74 (11.02)	8.29 (13.42)	1.20 (0.53)
W210	0.16 (0.03)	0.64 (0.02)	588.31 (22.98)	72.86 (19.91)	− 3.03 (0.81)
CCW	0.25 (0.04)	0.03 (0.03)	0.16 (0.04)	348.21 (18.69)	5.32 (0.53)
CONF	− 0.11 (0.03)	0.08 (0.03)	− 0.13 (0.03)	0.30 (0.03)	0.89 (0.03)

*BW* birth weight, *W90* weight at 90 days, *W210* weight at 210 days, *CCW* cold carcass weight, *CONF* conformation

The results for the residual variances, covariances and correlations are in Table 8.

**Table 8:** Posterior mean (and posterior deviation) of the residual variances (diagonal), covariances (upper diagonal) and correlations (lower diagonal) within the recursive model in the third scenario

	BW	W90	W210	CCW	CONF
BW	8.93 (0.07)	–	–	–	–
W90	–	436.48 (5.67)	432.36 (8.33)	–	–
W210	–	0.59 (0.01)	814.82 (13.08)	–	–
CCW	–	–	–	591.56 (13.12)	8.27 (0.36)
CONF	–	–	–	0.43 (0.01)	0.61 (0.02)

*BW* birth weight, *W90* weight at 90 days, *W210* weight at 210 days, *CCW* cold carcass weight, *CONF* conformation

**Fig. 4:**
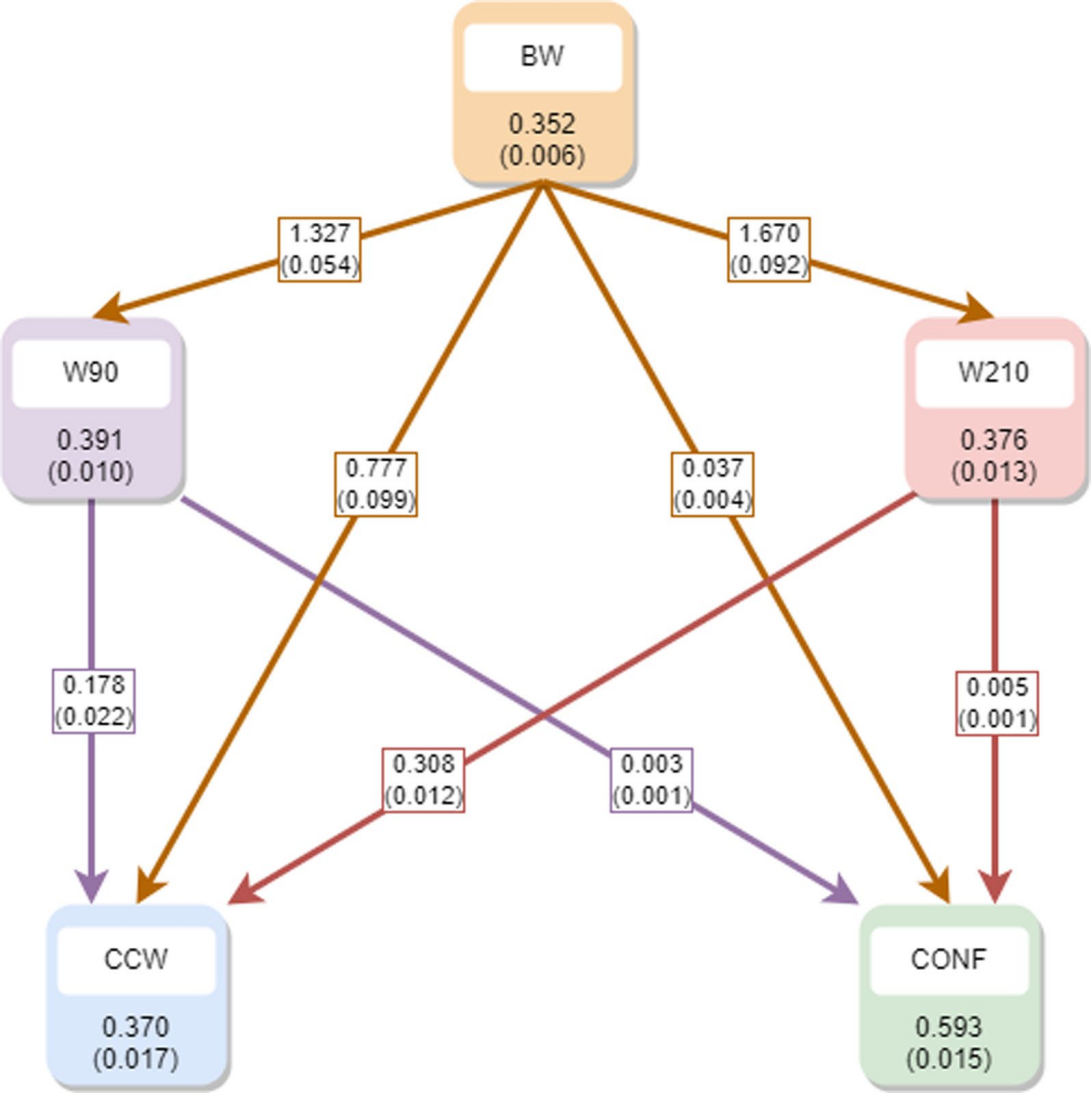
Posterior mean (and standard deviation) of the heritabilities (within the squares) and the recursive parameters (at the arrows) within the recursive model in the third scenario. *BW* birth weight, *W90* weight at 90 days, *W210* weight at 210 days, *CCW* cold carcass weight, *CONF* conformation

Finally, the posterior mean (and standard deviations) of the heritabilities and recursive parameters within the recursive model in the third scenario are displayed in Fig. 4.

It is important to emphasize that the heritabilities, as well as the covariances and correlations between CCW and CONF, remain unchanged from those obtained in the second scenario. This consistency arises because these estimates are conditioned on BW, W90, and W210, in spite of the inclusion of an additional recursive relationship between BW and W90, as well as W210.

## Discussion

We adopted the strategy proposed by Varona and Gonzalez-Recio [12] to obtained estimated variance components for recursive models, which suggests the use of the LDL′ or block-LDL′ transformations based on the estimates from a standard multiple trait model (SM). This approach allowed us to derive insights from up to three recursive models, all stemming from the output of a single SM model. Importantly, we conducted our analysis on a database with an uneven amount of information across the analysed traits.

The feasibility of this procedure hinges on the fundamental equivalence between the likelihood of the SM model and the recursive mixed model (RM). The only prerequisite for implementing the LDL′ or block-LDL′ transformations is that the likelihood remains identical. This condition is met when all pairs of traits are linked through either a recursive parameter or a residual covariance. If additional constraints beyond those necessary for identifiability are imposed in a RM (for instance, setting the recursive parameter and the residual covariance between a pair of traits to zero), the likelihoods of the SM and RM models diverge. They can then be compared using various goodness-of-fit tests.

In summary, the LDL′ or block-LDL′ transformations obviate the need to reanalyze data for each recursive model. Consequently, this approach enables multiple interpretations or breeding strategies to be derived from a single inference process. Moreover, handling missing data in recursive models, particularly when the independent traits are missing, presents challenges that can be avoided, as inference under several recursive models can be achieved through the SM. In this example, we have implemented a Bayesian analysis through a Gibbs sampler, but the same methodology can be used from the restricted maximum likelihood (REML) estimates obtained from any standard software. The primary advantage of using Markov chain Monte Carlo (McMC) is the ability to make inferences about the posterior distribution of recursive model parameters.

## Conclusions

The results of this study show that the LDL′ or block-LDL′ transformations, when applied to variance component estimates derived from a multiple-trait mixed model, enable making inferences across various sets of recursive models, with the only requirement being likelihood equivalence. Moreover, these transformations simplify the treatment of missing data when performing inference in the context of recursive models.

## Data Availability

The datasets analysed during the current study are available from the corresponding author upon reasonable request to the corresponding author.
